# *Cutibacterium acnes* regulates the epidermal barrier properties of HPV-KER human immortalized keratinocyte cultures

**DOI:** 10.1038/s41598-020-69677-6

**Published:** 2020-07-30

**Authors:** Beáta Szilvia Bolla, Lilla Erdei, Edit Urbán, Katalin Burián, Lajos Kemény, Kornélia Szabó

**Affiliations:** 1 Department of Dermatology and Allergology, University of Szeged, Szeged, Hungary; 2 HCEMM-SZTE Skin Research Group, Szeged, Hungary; 3 Department of Public Health, University of Szeged, Szeged, Hungary; 4 Institute of Clinical Microbiology, University of Szeged, Szeged, Hungary; 5 MTA-SZTE Dermatological Research Group, Szeged, Hungary

**Keywords:** Molecular biology, Skin diseases, Microbiome

## Abstract

Our skin provides a physical barrier to separate the internal part of our body from the environment. Maintenance of complex barrier functions is achieved through anatomical structures in the skin, the stratified squamous epithelium specialized junctional organelles, called tight junctions (TJs). Several members of our microbial communities are known to affect the differentiation state and function of the colonized organ. Whether and how interactions between skin cells and cutaneous microbes, including *Cutibacterium acnes* (*C. acnes*), modify the structure and/or function of our skin is currently only partly understood. Thus, in our studies, we investigated whether *C. acnes* may affect the epidermal barrier using in vitro model systems. Real-time cellular analysis showed that depending on the keratinocyte differentiation state, the applied *C. acnes* strains and their dose, the measured impedance values change, together with the expression of selected TJ proteins. These may reflect barrier alterations, which can be partially restored upon antibiotic–antimycotic treatment. Our findings suggest that *C. acnes* can actively modify the barrier properties of cultured keratinocytes, possibly through alteration of tight cell-to-cell contacts. Similar events may play important roles in our skin, in the maintenance of cutaneous homeostasis.

## Introduction

One of the most important properties of our skin is the complex barrier it provides to separate the internal part of our body from the environment, limiting contact with harmful chemicals, microbes, allergens and radiation^1–3^. The major building blocks of the skin barrier are the keratinocytes, which are capable of recognizing the ever-changing environmental conditions and mounting appropriate responses to maintain the integrity of the human body^4,5^.

Maintenance of complex barrier functions is achieved through anatomical structures in the skin. The stratified squamous epithelium is the uppermost skin layer that contains live keratinocytes and contains specialized junctional organelles, called tight junctions (TJs), which are localized between the cells of the second and third layer of the stratum granulosum^6^. TJs provide intimate links between adjacent cells and play major roles in establishing the epidermal barrier, as well as act as important determinants of transepidermal transport^7–9^. The complex, multi-protein structure of TJs includes more than 40 proteins^10,11^. Claudin (CLDN) protein family members are some of the most important TJ components, as they are critical for the regulation of barrier functions, including permselectivity, which determines the size, ionic charge and electric resistance of molecules that may be transported through the barrier^12,13^.

Keratinocytes are also in constant contact with various members of the cutaneous microbiota. One of the most well-known members of this community is the *Cutibacterium acnes* (*C. acnes*) bacterium, which, beginning with puberty, is a dominant species and preferentially inhabits sebum-rich skin regions^14,15^. Current research is elucidating a very interesting, mutualistic relationship between skin cells and this bacterium. The human skin offers a permissive environment for the colonization of *C. acnes* by providing nutrients, moisture, attachment sites and optimal temperature for the growth of the bacterium. *C. acnes*, in return for these available resources, produces a hostile environment for other, potentially pathogenic microbes^16,17^. As a result, *C. acnes* is now viewed as an important factor in the establishment and maintenance of epidermal homeostasis^18^.

Keratinocytes recognize the presence of *C. acnes* in their environment through the interaction of pattern recognition receptors (PRRs) expressed by the human cells and conserved molecules produced by microbes^19,20^. Consequently, innate immune and inflammation activation occurs, and autophagy may be induced^19–21^. Even though *C. acnes* is an important commensal bacterium, it can become an opportunistic pathogen during puberty as a result of microbial dysbiosis, in which it participates in the pathogenesis of a common inflammatory skin disease, acne vulgaris^22^. It has long been appreciated that immune and inflammatory events are crucial for acne lesion formation, and *C. acnes* may actively participate in these events; however, the exact sequence of pathogenic molecular events is still not known^23^.

Several members of our microbial communities are known to affect the differentiation state and function of the colonized organ. These functions are especially well described for the gut microbiota, which aids the differentiation and the development of anatomically mature, fully functioning gastrointestinal tract^24,25^. Whether and how interactions between skin cells and cutaneous microbes, including *C. acnes,* modify the structure and/or function of our skin is currently only partly understood. Thus, in our studies we investigate the effect of *C. acnes* on properties of the epidermal barrier and have found that this bacterium, similarly to other commensal microbes, also has a profound effect on these cutaneous functions. Our data also indicate that acne pathogenesis is possibly even more complex than was previously suspected, and, in addition to immune and inflammatory changes, altered barrier properties may also contribute to the disease of acne.

## Results

### Effect of *C. acnes* on the integrity of in vitro keratinocyte cultures

**Figure 1:**
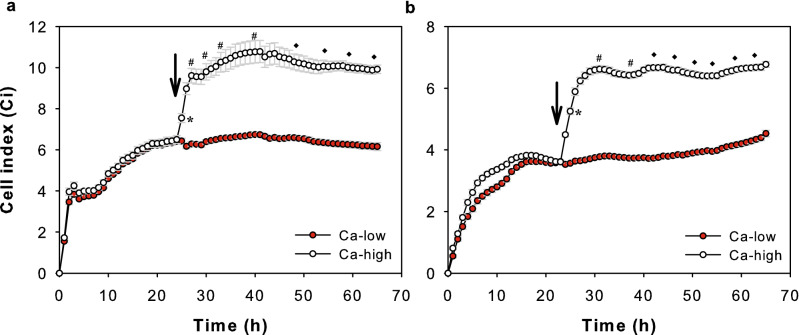
High Ca^2+^ concentration leads to elevated Ci values of NHEK and HPV-KER cultures. Confluent NHEK (**a**) and HPV-KER (**b**) monolayer cultures were incubated in standard KSFM media. After 24 h (marked with an arrow), the extracellular Ca^2+^ concentration was raised, leading to marked Ci increases. Representative image of three independent experiments. Data points are measured in every 4 h. Time points are the mean ± SEM. Statistical analysis with Student’s t-test, *p < 0.05, ^#^p < 0.005, ♦p < 0.0005.

Ohmic resistance and impedance measurements across a wide spectrum of frequencies are considered as good indicators of the integrity of cellular barriers^26–29^. Thus, we investigated how different culturing conditions affect the measurable impedance (Z), and the calculated Cell index (Ci) values of confluent NHEK (Fig. 1a) and HPV-KER (Fig. 1b) cultures using real-time cellular analysis, and interpreted the changes as alterations in barrier properties of the in vitro monolayer cultures^27–29^. We allowed the cells to form confluent monolayers using basal KSFM and then raised the concentration of extracellular Ca^2+^, which is known to induce keratinocyte differentiation^30^. We found that, after an initial growth phase, Ci values reached a plateau as the cultures became confluent and contact-inhibited. Replacing the media with fresh media containing high Ca^2+^ concentrations (Ca-high) induced a marked and immediate increase in Ci compared to samples that were only contact inhibited and maintained in low Ca^2+^ media (Ca-low). NHEK and HPV-KER cultures behaved similarly, suggesting that the latter cells may be used as a model to analyse keratinocyte barrier functions in monolayer cultures (Fig. 1a,b). These findings also agree with the available literature data and suggest that high extracellular Ca^2+^ concentration leads to the stabilization of keratinocyte barriers. In addition, the capability of the xCELLigence RTCA system for real-time monitoring of these properties was confirmed^31,32^.

**Figure 2:**
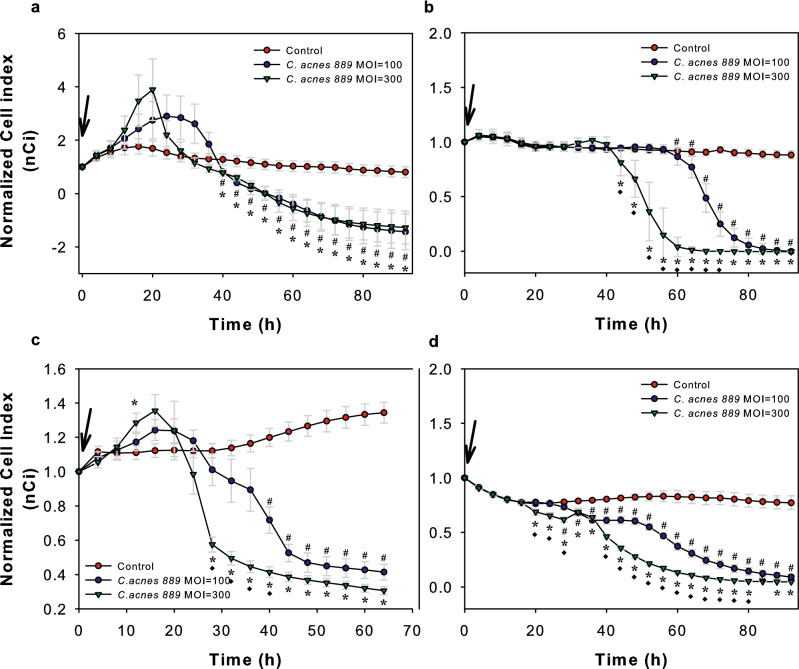
Co-culturing HPV-KER and NHEK keratinocyte monolayers with *C. acnes* 889 strain leads to dose-dependent nCi changes. Ca-low and Ca-high NHEK (**a**, **b**, respectively) and HPV-KER (**c**, **d**, respectively) keratinocyte monolayers were established and co-cultured with *C. acnes* 889 strain (0 h time point, marked with an arrow) in different doses (MOI = 100, 300). Addition of the bacterium induced dose-dependent nCi changes in all cultures. (For detailed description, see the corresponding text.) Data points are measured in every 4 h, representing the mean ± SEM. Statistical analysis with one-way ANOVA, post-hoc Tukey test, p < 0.05, alpha = 0.05, # control vs.* C. acnes 889,* MOI = 100, *control *vs. C. acnes 889*, MOI = 300, ♦ *C. acnes 889*, MOI = 100 *vs. C. acnes 889,* MOI = 300.

We used the same experimental setup to analyse how the addition of *C. acnes* to the Ca-low and Ca-high NHEK and HPV-KER cultures affected keratinocyte barriers. For this, *C. acnes 889* strain (MOI = 100, 300) was added and nCi values were recorded in every 4 h for 95 h (Fig. 2).

In Ca-low NHEK (Fig. 2a) and HPV-KER (Fig. 2c) cultures, we first observed a rapid and transient increase of nCi values, suggesting improved barrier properties of the cultures. Detailed analysis of these changes showed that the extent and exact shape of the curves depended on the MOI of *C. acnes* applied (Supplementary Fig. 1).

The peak in nCi was followed by a drop in the Ca-low cultures, suggesting that, after reaching a threshold, continuously growing bacterial cells may have deleterious effects on the barrier properties of keratinocyte monolayers. We also repeated the experiments on differentiated, Ca-high NHEK and HPV-KER monolayers. In these cell cultures only the dose-dependent decrease of nCi values were detected, independent of the used cell type and *C. acnes* MOI (Fig. 2b,d).

For determining strain-specific effects, we repeated the experiment using different *C. acnes* strains and HPV-KER monolayers. Similar results were detected, suggesting that the observed nCi changes may be independent of the applied strains (Supplementary Fig. 2).

When experiments were repeated using heat-killed *C. acnes*, no significant changes in nCi were detected, suggesting that the impedance changes observed in previous experiments depended on the presence of live bacteria (Supplementary Fig. 3a–c). This prompted us to test whether PA, an important molecule produced during *C. acnes* metabolism, produced similar effects. Indeed, treatments with high concentrations of PA resulted in a transient increase in nCi, followed by marked decreases (Supplementary Fig. 4).

Apart from the barrier properties, Ci changes may reflect differences in the number or in the specific dimensions of cells attached to the electrodes. To determine the exact nature of the *C. acnes*-induced cellular events, we monitored the effect of the bacterium on the number of cells both in the Ca-low and Ca-high HPV-KER cell cultures. No significant change in cell number was detected using a trypan blue exclusion assay (Supplementary Fig. 5a,b).

**Figure 3:**
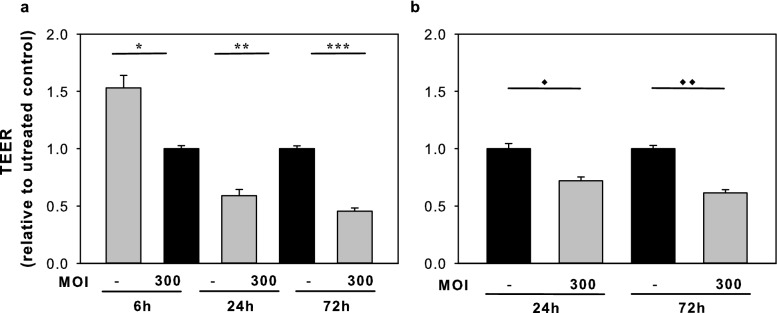
Transepithelial electrical resistance (TEER) analysis of *C. acnes 889* co-cultured Ca-low (**a**) and Ca-high (**b**) HPV-KER monolayer cultures. *C. acnes 889* strain **(**MOI = 300) induced marked TEER in the HPV-KER monolayer cultures, and these results were confirmed with real-time cellular analysis experiments. (Each treatment was performed in three technical replicates. Data points are the mean ± SEM. Statistical analysis with Student’s t-test, * p < 0.05, **p < 0.005, ***p < 0.00005, ♦ p < 5 × 10^–15^, ♦♦ p < 5 × 10^–24^).

We also performed TEER measurement, a widely accepted quantitative technique to analyse tight junction dynamics^26^. Selected time-points (24 and 72 h) were chosen, based on our real-time investigation, and TEER measurement was performed using untreated samples as a control and samples treated with *C. acnes* (MOI = 300) in both Ca-low and Ca-high HPV-KER monolayers. Our results indicate that the TEER resistance values first increased in the Ca-low cultures, followed by a decrease in both models in the presence of the bacterium, similarly to what we found in the xCELLigence analysis (Fig. 3).

Altogether, these results strongly suggest that *C. acnes* is able to influence the state of the in vitro cultured keratinocyte barrier. This effect requires the presence of metabolically active, live bacteria, and the extent of the changes are dependent on the bacterial dose.

### The effect of *C. acnes* 889 strain on the expression of selected TJ proteins in HPV-KER cultures

**Figure 4:**
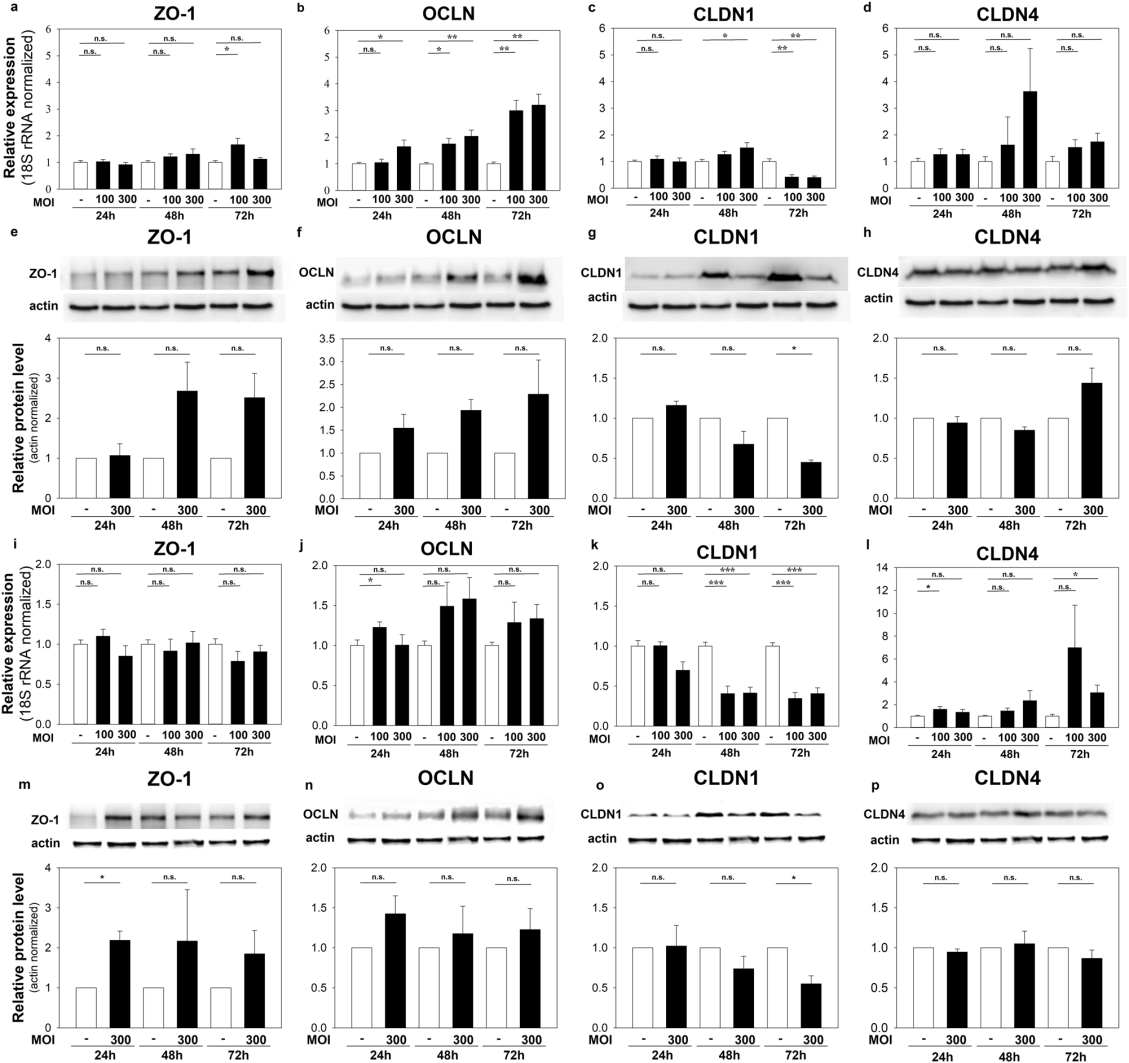
mRNA- and protein-expression changes of selected TJ proteins in Ca-low (**a**–**h**) and Ca-high (**i**–**p**) HPV-KER monolayer cultures co-cultured with *C. acnes* 889 strain. ZO-1 (**a**,**e**,**i**,**m**), OCLN (**b**,**f**,**j**,**n**), CLDN1 (**c**,**g**,**k**,**o**) and CLDN4 (**d**,**h**,**I**,**p**) mRNA and protein expression changes were monitored by real-time RT-PCR and western blot analysis. For detailed explanation, see the corresponding text. (mRNA data corresponds to the average of three parallel experiments, where each treatment was performed in three replicates. Western blot data shows a representative experiment. Detailed description can be find in Supplementary Figs. 6 and 7. Data points represents the average ± SEM. Statistical analysis with Student’s t-test, not significant (n.s.), *p < 0.05, **p < 0.005, ***p < 0.00005. The representative western blot and the graph corresponds to the average of three independent experiments).

To study the cellular changes leading to *C. acnes*-induced nCi alterations, we treated Ca-low and Ca-high HPV-KER cultures with *C. acnes 889* strain (MOI = 100, 300) and analysed the expression of selected genes encoding important TJ components: claudin 1 and 4 (CLDN1, CLDN4), occludin (OCLN) and zonula occludens 1 (ZO-1). mRNA expression studies were detected with real-time RT-PCR, and protein expression levels were detected with western analysis. ZO-1 mRNA levels did not markedly change, and OCLN expression increased upon bacterial treatment (Fig. 4a,b,i,j). The expression of the two claudins exhibited opposing changes: CLDN1 levels decreased in both models, while CLDN4 increased in the Ca-low cultures (Fig. 4c,d,k,l).

We found that, upon the addition of the bacterium, ZO-1 levels increased markedly in the Ca-high cultures (Fig. 4m), and a small, but consistent elevation was detected in the Ca-low HPV-KER cultures (Fig. 4e). OCLN expression increased slightly (Fig. 4f,n), whereas CLDN1 protein levels decreased in both cultures (Fig. 4g,o). CLDN4 levels also increased but only in the Ca-low cultures 72 h after treatment (Fig. 4h,p).

Our findings suggest that *C. acnes* induces the expression changes of selected TJ structural proteins.

### The effect of *C. acnes* 889 strain on the expression of selected TJ proteins in OS models

To determine whether the *C. acnes*-induced cellular changes were specific to monolayer cultures, we also investigated the effect of *C. acnes* on the expression of selected TJ structural components in ex vivo, three-dimensional OS cultures. In these experiments, we treated the upper, epidermal side of the skin samples with live *C. acnes* 889 strain. We collected samples at 24, 48 and 72 h after treatment, and IHC staining was performed to monitor the mRNA and protein expression changes.

**Figure 5:**
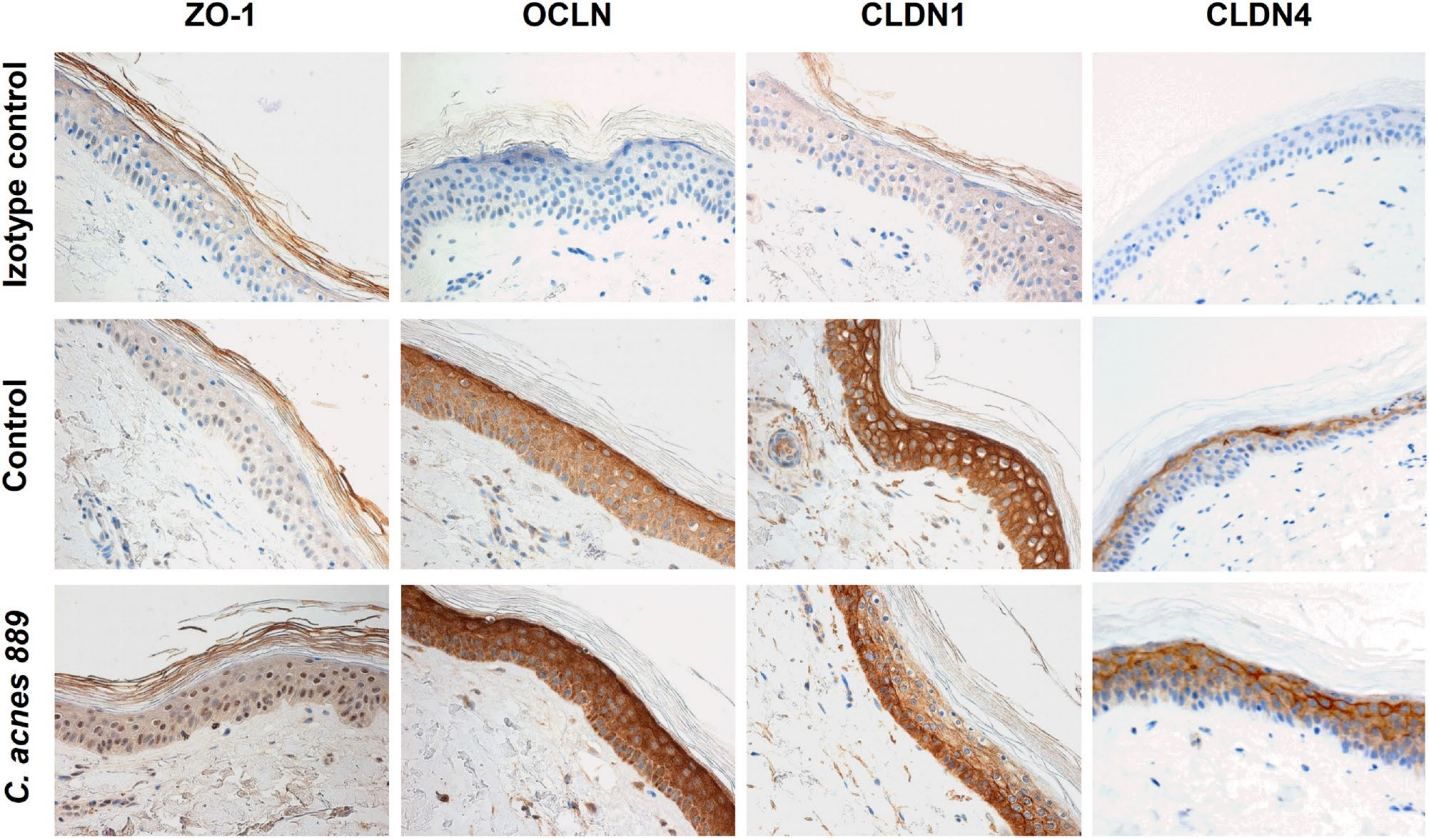
TJ protein expression changes in OS models upon treatment with *C. acnes* 889 strain. Full-thickness skin biopsy samples were treated with live *C. acnes* strain 889 (1.5 × 10^7^ bacteria/sample) for 72 h. Paraffin-embedded sections were analysed by IHC staining. We detected marked changes in expression and tissue localization for all the analysed TJ proteins 72 h after bacterial treatment. (Representative image of three independent experiments).

We found that ZO-1 and OCLN protein levels increased in all epidermal layers in the presence of *C. acnes* 889 strain. CLDN1 expression, which was strongly expressed throughout the epidermis in the untreated control samples, decreased everywhere except the basal layer. In contrast, CLDN4, which was restricted to the *stratum granulosum* in control samples, also appeared in the lower layers of the epidermis in the presence of the bacterium (Fig. 5). These findings suggest that the abundance as well as the localization of selected TJ proteins change in the presence of the bacterium.

### LY penetration assay of HPV-KER monolayer cultures and OS models

To analyse the functional consequences of the observed nCi changes, LY penetration assay was performed on the Ca-high HPV-KER monolayers and OS models.

Ca-high cultures were first treated with *C. acnes* 889 strain (MOI = 300) for 24 or 72 h in a transwell system; controls were treated with PBS alone. We chose these time points based on the results of our previous TEER and xCELLigence experiments. LY dye was added to the upper chamber, and the penetration assay was carried out for 30 min. Samples from the cell supernatant collected from the lower chambers were subjected to fluorimetry.

**Figure 6:**
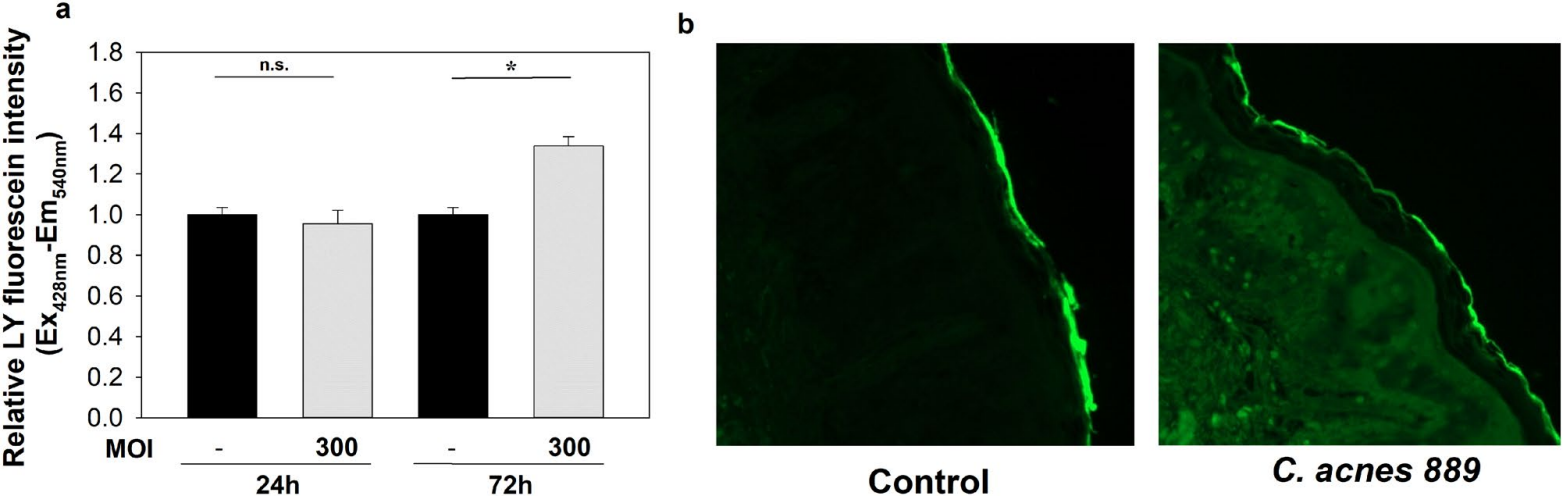
LY dye penetration experiments. Co-culturing with *C. acnes* 889 strain for 72 h leads to increased LY dye penetration in Ca-high HPV-KER monolayer cultures (**a**) and OS models (**b**). (The graph represents the average of three technical replicates. Relative LY fluorescence intensity was compared to the time-matched untreated control samples. Data points represent the average ± SEM, statistical analysis with Student’s t-test, not significant (n.s.), p < 0.0001. Panel b is a representative of three independent experiments).

We found that fluorescence intensities increased in treated samples compared to controls, indicating LY dye penetration by 72 h after treatment. No differences were detected in the 24-h samples (Fig. 6a).

We also repeated the dye penetration experiments in the OS models to confirm that the observed barrier changes were not specific to monolayer cultures. *C. acnes* 889 strain or PBS was pipetted to the upper, epidermal side of the OS samples and samples were incubated for 72 h before the addition of LY, and dye penetration was visualized by fluorescent microscopy.

As observed for the monolayer cultures, LY dye penetration increased in the presence of high-MOI *C. acnes* 889 treatment, as indicated by the appearance of increased, diffuse fluorescent signal in the sub epidermal skin regions (Fig. 6b).

### Antibiotic treatment of *C. acnes* co-cultured HPV-KER cells leads to the partial reversal of decreased nCi values

**Figure 7:**
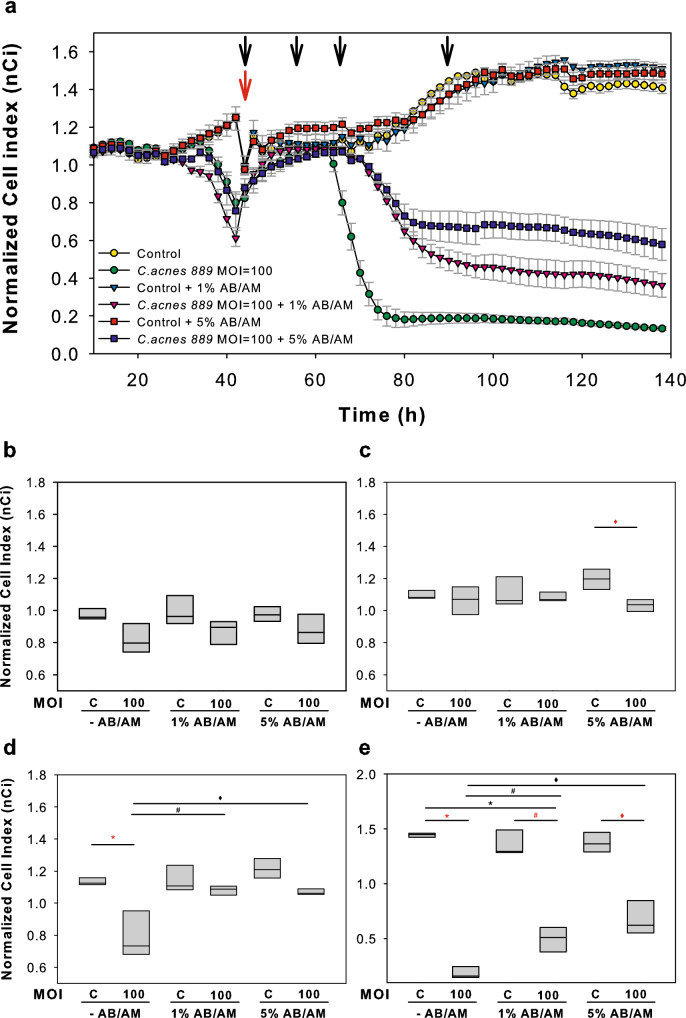
Antibiotic treatment of *C. acnes* 889 co-cultured HPV-KER monolayers. (**a**) Limiting the *C. acnes* bacterium with the addition of AB/AM solution transiently restores the nCi values in a dose-dependent manner. 0 h represents the time of *C. acnes* 889 treatment. (**b**–**e**) Box plot representation of selected time points after AB/AM treatment (red arrow) at 0 (**b**), 14 (**c**), 24 (**d**) and 48 h (**e**) (marked with black arrows). (Representative image of three parallel experiments, where each treatment was performed in five technical replicates. Data points represent the mean ± SEM. Statistical analysis with one-way ANOVA, post-hoc Tukey test, p < 0.05, alpha = 0.05, red * control vs.* C. acnes 889,* MOI = 100, red # control + 1% AB/AM vs.* C. acnes 889,* MOI = 100 + 1% AB/AM, red ♦ control + 5% AB/AM vs. *C. acnes 889,* MOI = 100 + 5% AB/AM, * *C. acnes 889,* MOI = 100 vs.* C. acnes 889*,MOI = 100 + 1% AB/AM, # *C. acnes 889,* MOI = 100 vs.* C. acnes 889,* MOI = 100 + 5% AB/AM, ♦ *C. acnes 889,* MOI = 100 + 1% AB/AM vs. *C. acnes 889* MOI = 100 + 5% AB/AM).

As many of the acne treatments currently available exhibit bacteriostatic and/or antibacterial properties, we were interested to see if decreasing the *C. acnes* activity and/or viability in co-cultures with the addition of AB/AM treatment reverses the bacterium induced nCi decreases we noted in previous experiments. To this end, Ca-low HPV-KER cultures were co-cultured with *C. acnes* 889 strain (MOI = 100), and the effects of the bacterium were continuously monitored in real time using the xCELLigence system. When the nCi values started to drop, we changed the culturing media to KSFM supplemented with 1% or 5% AB/AM solution. Decreasing the load of live bacteria in the co-cultures by the addition of AB/AM solution partially reversed the deleterious effect of *C. acnes* 889 strain and led to a marked increase of nCi values, characteristic of improved barrier functions of in vitro keratinocyte cultures (Fig. 7a–e).

## .Discussion

Although the cutaneous microbiota plays very important roles in the maintenance of the skin’s homeostasis, the interactions between human cells and the various microbes also generate many challenges for the body. Skin cells, especially keratinocytes, must tolerate the presence of these microbes, even though they are equipped with all the conserved molecular structures that stimulate PRRs^19,20^. Thus, the skin must maintain a proper barrier against potentially harmful pathogens while simultaneously allowing continuous crosstalk between beneficial microbes and cutaneous immune cells^33–35^. Such complex interactions require fast, dynamic epidermal-barrier functions. In our study we aimed to investigate whether and how the cutaneous microbiota, particularly the *C. acnes* bacterium affects the properties of the skin barrier.

Our interest led us to perform in vitro studies using two keratinocyte monolayer cultures (NHEK, HPV-KER) as a model system for skin with real-time impedance measurement-based studies. These methods are widely used for the detection of cellular integrity and barrier changes of in vitro monolayer cultures^26,36^. Using the gathered data, together with the results we obtained by analysing full thickness OS models, we propose a possible model, how *C. acnes* may affect the cutaneous barrier in the healthy skin and during acne pathogenesis.

We found that *C. acnes*, a prominent member of the skin microbiota, had a complex effect on these cells. In less differentiated, Ca-low cultures, co-culturing with live bacterium induced rapid, transient nCi increases, which are characteristic of improved barrier properties. These changes were strain-specific and depended on the applied bacterial MOI. A fast growing pathogenic strain, *C. acnes 889*; IA, induced rapid and marked nCi increases compared to the *C. acnes 6609* strain, which exhibits slower growth^37^. The effect of strain *6609*, which was originally isolated from the facial region of a healthy subject, exhibited a less pronounced, but sustained effect.

*C. acnes* is not the only microbe that may strengthen the skin barrier. Ohnemus and colleagues reported similar results with another member of the skin microbiota, *Staphylococcus epidermidis*^38^. The observed phenomena are also very similar to those observed for the intestinal microbiota, which also aids the differentiation and the development of the anatomically mature, fully functioning gastrointestinal tract^24,25^.

When left long enough in contact with the cells, continuously growing *C. acnes* strains gradually exhibited deleterious effects on the integrity and barrier properties of keratinocyte monolayer cultures, as indicated by the marked decrease in nCi values. This effect was dependent on the dose of live bacteria applied. The decrease in nCi was independent of cell differentiation. In addition, the change in nCi was not caused by keratinocyte death, as no major changes were detected in the number of live cells in the HPV-KER monolayer cultures.

nCi changes were only observed when we co-cultured the keratinocytes with live *C. acnes* strains. It is possible that these changes are not solely caused by conserved microbial structural components, but these changes might also involve a variety of special compounds that are generated by bacterial metabolism. *C. acnes* produces short chain fatty acids as a result of anaerobic fermentation, enzymes (*e.g.,* lipases) and other biologically active molecules, that impact important regulatory functions of a number of human cell types^39^. Identifying which of the many molecules are necessary for the regulation of cutaneous barrier functions requires further investigation. As an initial effort, we analysed the effect of PA, a special metabolic product of this bacterium that is linked to bacterial growth and metabolism^37^. We found that PA may be an important factor: Ca-low cultures treated with high doses of PA exhibited clear, transient Ci changes that were comparable to what we observed with live *C. acnes* strains.

Next, we aimed to see whether the detected resistance changes were correlated with changes in in vitro barrier integrity. Treatment with LY, a large molecular weight, fluorescently labelled compound that cannot freely permeate lipophilic barriers, clearly indicated that the nCi decrease could be the consequence of bacterium-induced deterioration of the integrity of the HPV-KER keratinocyte culture. This effect was not specific to monolayer cultures: similar results were found when the experiments were repeated in OS models, suggesting that *C. acnes* may also regulate cutaneous barrier properties, tightness and paracellular transport properties of the epidermis, even in a tissue environment.

Multiple different claudins, which are also essential proteins in TJ complexes, are present simultaneously in a given cell type, and the expression of various family members are highly context-dependent, controlled both spatially and temporarily. Many other factors, including tissue type, age, differentiation state, external and intracellular signals and stimuli, are involved in their regulation^40,41^. The claudin content of a given cell is not stable and can change significantly in a matter of hours. Such continuous molecular remodelling, called switching, is an important process that allows the epidermal barrier to adapt to the ever-changing external and internal environments^42^. TEER and the associated paracellular permeability are thought to depend on the profile of different claudins expressed in a given cell type at a given point in time ^12^. In the skin, keratinocytes express several different claudin family members, but the most abundantly present proteins are CLDN1, CLDN 4 and CLDN 7^43^. It is interesting that apart from keratinocytes, sebocytes also express the same claudins. They play important roles in the regulation of a permeability barrier, and possibly also during the holocrine secretion process in these glands^44^. Whether and how *C.acnes* affects the expression of these claudins, and through that sebaceous gland functions requires further investigations.

Immunostaining of characteristic TJ proteins has been used to gain insight into the integrity of different barriers^26^. In full-thickness skin organ cultures, ZO-1 and OCLN protein levels increased in all epidermal layers following treatment with *C. acnes* 889 strain, and we observed similar changes in monolayer cultures. CLDN1 and CLDN4 expressions exhibited opposite changes: while CLDN1 levels mostly decreased in the presence of the bacterium, CLDN4, which was restricted to the *stratum granulosum* in control OS samples, simultaneously appeared in the lower layers of the epidermis. These protein expression changes correlated well with changes in mRNA expression, especially in the case of OCLN, CLDN1 and CLDN4. These findings suggest that TJ protein levels and their localization change in the presence of the bacterium, and their regulation possibly involves transcriptional and post-transcriptional effects, as well^45^.

CLDN1 is a key determinant of epidermal barrier functions in human and mouse epidermis^46,47^. A strong positive correlation between epidermal integrity and CLDN1 expression levels was found in rodents. CLDN1-deficient mice exhibit signs of hyperkeratosis and acanthosis with aging, suggesting that abnormal regulation of this protein affects multiple cellular mechanisms^48^. Reduced CLDN1 levels appears to be a common theme in different chronic inflammatory skin diseases, including psoriasis, atopic dermatitis and rosacea^47–49^.

CLDN4 levels increased when keratinocytes and cells of the OS model were co-cultured with *C. acnes* 889 strain. CLDN4 is also an important molecule of the epidermal barrier, and the decrease its expression in in vitro cultured keratinocytes has been associated with barrier defects. CLDN4 is considered a tightening claudin, and our results are compatible with the idea that its upregulation presents a compensatory mechanism to restore barrier functions caused by decreased CLDN1 expression levels^7,50^.

Opposing regulation of CLDN1 and CLDN4 has been reported before, and CLDN1/CLDN4 switching may be an important mechanism to regulate the epidermal barrier during inflammatory skin conditions^47,51^.

Overall, through the induction of mostly TLR-dependent innate immune and inflammatory changes and the production of bioactive molecules, the *C. acnes* bacterium may induce innate immune and inflammation activation and autophagy, as well as altering the differentiation state of skin cells and epidermal barrier functions^19–21,52^.

In our experiments, the extent of barrier changes in keratinocyte cultures appeared to be strain- and dose-dependent: faster growing *C. acnes strains* (889 and ATCC 11828) exhibited more pronounced changes. These results confirm our earlier findings, suggesting that, in parallel with bacterial growth, the pathogenic potential of *C. acnes* increases^37,52^. This conclusion contradicts findings that the amount of the bacterium does not differ in control and acne skin^53,54^. The reason for these discrepancies may be complex. Only a limited number of follicles are inflamed at any given time, and *C. acnes* may not reach the skin surface as it becomes trapped inside the follicles, especially when present in a biofilm form. Once inside the pilosebaceous unit, its density may rapidly increase during lesion development.

Topical and systemic drugs are widely used for acne treatment^55^. We tested whether reducing the presence of viable *C. acnes* using antibiotics in the HPV-KER co-cultures restores the barrier properties and found clear increases in the measured nCi values. Our results suggest that acne pathogenesis may be an even more complex event than previously suspected, and that, apart from the role of immune and inflammatory events, changes in cutaneous barrier properties can play important roles. Further experimental and clinical studies are necessary to determine whether therapeutic modalities restoring supplementary barrier together with conventional antibacterial treatments would enhance the healing of lesions.

## Materials and methods

### *C. acnes* strains and culture conditions

*C. acnes 889* (type IA) and *6609* (type IB) strains were cultured and stored as previously described in detail^20,37^.

### Keratinocyte cell cultures, organotypic skin models and *C. acnes* treatment

Normal human keratinocytes (NHEK) and ex vivo skin biopsies were obtained from skin specimens collected from the Plastic Surgery Unit of our department. Written, informed consent was obtained from the investigated individuals. The study was approved by the Human Investigation Review Board of the University of Szeged (PSO-EDAFN-002, 23 February 2015, Szeged, Hungary) and complied with the ethical standards of research in accordance with the Helsinki Declaration.

The human immortalized keratinocyte cell line HPV-KER^37^ and NHEKs were used for our experiments. Both cell types were cultured in keratinocyte serum-free medium (KSFM, Life Technologies, Carlsbad, USA) containing 1% antibiotic/antimycotic (AB/AM, Sigma Aldrich, St. Louis, MO, USA) solution and supplemented with epidermal growth factor and brain pituitary extract under standard laboratory conditions (37 °C in a humidified atmosphere containing 5% v/v CO_2_). Differentiation of confluent NHEK and HPV-KER cultures was induced with the addition of 1.7 mM CaCl_2_.

For generating the organotypic skin (OS) models, 1 cm^2^ whole-thickness skin samples were placed onto the nitrocellulose membrane of a 6-well insert, with the dermal surface placed on the membrane. Samples were cultured in an air–liquid interphase using Dulbecco’s Modified Eagle’s/F12 medium supplemented with 10% fetal bovine serum. *C. acnes* culture (15 μl, 10^9^ CFU/sample) was transferred to skin samples (epidermal surface) and further cultured for an additional 24, 48 or 72 h. Samples for immunofluorescent staining were fixed in 4% paraformaldehyde and embedded in paraffin. For PCR analysis, 6 mm punch biopsies were taken from the samples and the epidermis was separated from the dermis by incubating in dispase solution for 3 h at 37 °C. Epidermis samples were homogenized using the Ultra Turrax T8 homogenizer (IKA-WERKE). Total RNA was isolated using TRI-Reagent (Molecular Research Center; Cincinnati, USA).

### Real-time, label-free cellular analysis of keratinocyte cultures

The integrity and the barrier properties of HPV-KER and NHEK monolayer cultures were detected in real-time using an impedance-measurement based technology (xCELLigence, Real Time Cell Analyser system, ACEA Biosciences, San Diego, USA)^31,56^. For contact inhibited cultures, NHEK and HPV-KER cultures were grown to confluency on fibronectin-coated 96-well E-plates (Ca-low cultures). After 24 h of growth, cells were co-cultured with live *C. acnes* strains belonging to different phylogenetic groups within the species (*889, ATCC 11828, 6609*) at various multiplicity of infection (MOI). Each treatment was performed in five technical replicates. Impedance (Z) values were measured every 60 min (unless otherwise noted), from which a dimension-free cell index (Ci) was calculated (Ci = [impedance at time point n – background impedance without cells]/nominal impedance value). In the used confluent cultures, Ci values depend on the applied cell number, the cell–cell adhesion and the cell-surface interaction. In some experiments, normalized Ci (nCi) values were also determined (nCi = Ci values at time point n/Ci values at a selected time point, *e.g.,* time point of applied treatment). Ci and nCi were plotted as a function of time. Each data point represents the mean ± the standard error of the mean (SEM).

For the differentiated NHEK and HPV-KER (Ca-high) cultures, KC-SFM culturing media was supplemented with 1.7 mM CaCl_2_ after the cultures reached confluency. Forty-eight hours after treatment, the differentiated cultures were treated with *C. acnes* strains and nCi changes were monitored as described above.

The effect of propionic acid (PA) was determined using HPV-KER (Ca-low) cultures. After a confluent state was reached, PA was added to the cells at different concentrations, and nCi changes were monitored as described above.

Each treatment was performed in five technical replicates and the reported data points represent the mean ± SEM, unless otherwise noted.

### Western blot analysis of TJ proteins

Cells were scraped with a cell scraper and harvested by centrifugation. The pellets were resuspended in protein lysis buffer (20 mM HEPES, 150 mM KCl_2_, 1 mM MgCl_2_, 1 mM dithiothreitol, 5% TritonX-100, 10% glycerol, 0.1% NP-40) containing 1% protein inhibitor cocktail, 1% phenylmethylsulfonyl fluoride, 0.5% sodium dodecyl sulfate (all components from Sigma). Protein concentrations were measured with the BCA detection kit (Thermo Scientific, Waltham, MA, USA). SDS-PAGE was carried out using 50 µg protein sample, and proteins were transferred to a nitrocellulose membrane (Bio-Rad Laboratories, Hercules, CA, USA). Membranes were blocked with Tris-buffered saline solution (150 mM NaCl, 25 mM Tris, pH 7.4) containing 3% and 5% non-fat dry milk powder (Bio-Rad). Rabbit anti-human actin (Sigma-Aldrich) and anti-ZO-1 (Thermo Fisher Scientific) antibodies were diluted at 1:1,000, mouse anti-claudin 1 (Abnova), anti- occludin (Abnova) and anti-Claudin-4 (Thermo Fisher Scientific) were diluted at 1:500, and membranes were incubated with antibodies overnight at 4 °C. Anti-rabbit and anti-mouse IgG horseradish peroxides-conjugated (HRP-conjugated) secondary antibodies (SouthernBiotech) were applied, and the resulting bands were visualized using the C-DiGit Blot Scanner (LI-COR Biosciences, Nebraska, USA) or the Omega Lum G Chemidoc Imageing System (Aplegen, Inc, Pleasanton, CA, USA). Original western blots are provided as Supplementary Figs. 6 and 7.

### Total RNA isolation and real-time RT-PCR

Total RNA was isolated from the monolayer cultures using TRI-Reagent (Molecular Research Center; Cincinnati, USA). cDNA synthesis was performed using EvoScript cDNA Synthesis Kit (Roche), and changes in the expression of TJ genes (CLDN1, CLDN4, OCLN and ZO-1) were analysed by real-time RT-PCR using the Universal Probe Library system (Roche, Indiana, USA). Supplementary Table 1 lists the PCR protocols and primer sequences used. TaqMan Gene Expression Assays (Thermo Fischer Scientific, Massachusetts, USA) were used to detect tumor necrosis factor α (TNFα; HS00174128_m1) and CXC motif chemokine ligand 8 (CXC8; HS00174103_m1). All data were normalized to the 18S rRNA using the ΔΔC_t_ method before comparing to the time-matched untreated control samples. Supplementary table 1 lists the used primers and probes.

### Immunohistochemical staining

Paraffin-embedded OS samples were used to analyse changes in TJ protein levels after *C. acnes 889* treatment. A Bond-Max automated IHC/ISH stainer (Leica Biosystems, Wetzlar, Germany) was used for immunohistochemical (IHC) staining. The staining protocol is described here briefly. Six µm sections were cut, placed on glass slides, deparaffinized (dewax, 72 °C) and rehydrated. Subsequently, antigen retrieval was performed (10 mM citrate buffer, pH 6.0, 100 °C, 20 min). Primary antibodies against TJ proteins [CLDN1 (Abcam, Cambridge, UK), CLDN4 (Thermo Fischer Scientific, Massachusetts, USA), OCLN (Abnova, Taiwan), ZO-1 (Thermo Fischer Scientific, Massachusetts, USA)] were added to the samples at 1:100 dilutions (RT, 30 min), and also matching isotype controls [mouse IgG2a (Biolegend, CA, USA), rabbit poly IgG (Santa Cruz Biotechnology Inc, CA, USA) and mouse IgG1 κ (Biolegend, CA, USA)] which dilution rations were matched to the used primary antibodies concentrations. After primary antibody labelling, post primary steps were performed for 8 min at room temperature (RT), subsequently, the polymer step was performed (15 min, RT). Peroxidase blocking was carried out (3 min, RT). Washing steps were performed before each step. Samples were developed with DAB-Chromogen for 10 min and dyed with hematoxylin. After covering with coverslips, samples were analysed with a microscope (Carl Zeiss Microscopy GmbH, Munich, Germany) equipped with an AxioCam MRm camera.

### Transepithelial electrical resistance measurement

Confluent HPV-KER monolayer cultures were grown on 12 mm transwell inserts (pore size 0.4 µm, Corning, New York, USA) in KSFM media. Confluent Ca-low cultures were treated with *C. acnes* 889 strain (MOI = 300) and transepithelial electrical resistance (TEER) values were measured in Hank’s salt balanced solution (HBSS) using an Epithelial Volt/Ohm (TEER) Meter EVOM2 (World Precision Instruments, Sarasota, FL, USA) at 24 and 72 h after treatment.

For the Ca-high cultures, confluent, contact-inhibited keratinocyte cultures were further differentiated by the addition of Ca^2+^ (1,7 mM) to the KSFM culturing media and incubation for 72 h. TEER values were measured at 24 and 72 h after treatment with *C. acnes* 889 in HBSS buffer using an Epithelial Volt/Ohm (TEER) Meter EVOM2 (Sarasota, FL, USA).

### Trypan blue exclusion assay

HPV-KER cells were seeded on 12-well plates at a starting density of 300,000 cells/well. After 48 h, the cultures were co-cultured with various doses of *C. acnes* 889 strain (MOI = 100, 300). Samples were trypsinized and collected at 0, 24, 48 and 72 h after treatment, washed with phosphate-buffered saline (PBS) and stained with trypan blue dye (Sigma-Aldrich, St. Louis, Missouri, United States). Viable cells were counted using a hemocytometer.

### Lucifer yellow penetration assay

HPV-KER cells were seeded on porous 12-well plates (pore size 0.4 µm, Corning, New York, USA) at a density of 1.5 × 10^5^ cells/well using KSFM (Life Technologies, Carlsbad, USA). Ca-high cultures were established and treated with *C. acnes* 889 strain (MOI = 300) for 24 and 72 h. Lucifer yellow (LY) penetration experiments were carried out in HBSS containing 100 µM LY (Sigma-Aldrich, Saint Louis, Missouri, USA). HBSS-LY media was added to the upper chamber and samples were collected from the bottom chamber after 30 min of incubation using standard culturing conditions (5% v/v CO_2_, 37 °C). Fluorescence intensities were measured with a BMG FLUOstar OPTIMA Fluorescence plate reader (Gemini BV Laboratory, Netherland) and relative fluorescence intensities were calculated. Fluorescence intensities were normalized to the time-matched untreated control values.

In the case of the OS models, *C. acnes* 889 strain (15 μl, 10^9^ CFU/sample) was pipetted onto the upper, epidermal part of the skin samples, which were then incubated for 72 h. For the transport experiments, LY diluted to 1 mM LY with PBS (15 μl/sample) was applied to the top. After 3 h, skin samples were collected using 6 mm punch biopsies and embedded in Shandon Cryomatrix (Thermo Fischer Scientific, Massachusetts, USA) for frozen sectioning. Sections (6 µm) were cut from the samples, and LY dye penetration was analysed using a Zeiss Axio Imager Z1 fluorescence microscope (Carl Zeiss Microscopy GmbH, Munich, Germany) equipped with an AxioCam MRm camera.

### Antibiotic treatment of C. acnes co-cultured HPV-KER monolayers

The effect of antibiotic treatment of *C. acnes* co-cultured HPV-KER monolayers was observed in real time using the RTCA system. HPV-KER cells were plated at a density of 30,000 cells/well in fibronectin coated 96-well E-plates. After the cultures reached confluency, they were treated with *C. acnes 889* strain (MOI: 100) and, 44 h later, 1% or 5% AB/AM was added to the cultures. Each treatment was performed in five technical replicates. Impedance values were measured every 60 min for 120 h, and a dimension-free cell index (Ci) was calculated from the data. Ci (average of the technical replicates) tracings were normalized to values recorded at the addition of the bacterium to the cultures, and the resulting nCi values were plotted.

### Statistical analysis

Unless otherwise noted, all the data are presented as mean ± SEM for three independent experiments. For xCELLigence analysis, treatment was performed in at least five times. For real-time RT-PCR and trypan blue exclusion experiments, each treatment was performed at least three times. For western blot analysis and IHC staining, each condition was repeated once in each independent experiment. Data were compared using paired Student’s t-test and one-way ANOVA with post-hoc Tukey-test. A probability value of less than 0.05 was considered significant.

## Supplementary information

Supplementary information.

## References

[1] Proksch E, Brandner JM, Jensen J-M (2008). The skin: An indispensable barrier. Exp. Dermatol..

[2] Jensen JM, Proksch E (2009). The skin’s barrier. G. Ital. Dermatol. Venereol..

[3] Kemény L, Nagy N, Csoma Z, Szabó K, Eros G (2016). Pharmacological targeting of the epidermal barrier. Curr. Pharm. Des..

[4] Denda M (2016). Keratinocytes at the uppermost layer of epidermis might act as sensors of atmospheric pressure change. Extreme Physiol. Med..

[5] Denda M, Nakanishi S (2020). Epidermal keratinocyte sensing and processing of environmental information together with the brain’s simulation and prediction abilities helped to enable *Homo sapiens’* evolutionary success. Adv. Anthropol..

[6] Yoshida K (2013). Functional tight junction barrier localizes in the second layer of the stratum granulosum of human epidermis. J. Dermatol. Sci..

[7] Brandner JM (2016). Importance of tight junctions in relation to skin barrier function. Curr. Probl. Dermatol. (Switz.).

[8] Yokouchi M (2016). Epidermal cell turnover across tight junctions based on Kelvin’s tetrakaidecahedron cell shape. Elife.

[9] Yokouchi M, Kubo A (2018). Maintenance of tight junction barrier integrity in cell turnover and skin diseases. Exp. Dermatol..

[10] Paris L, Tonutti L, Vannini C, Bazzoni G (2008). Structural organization of the tight junctions. Biochim. Biophys. Acta Biomembr..

[11] Chiba H, Osanai M, Murata M, Kojima T, Sawada N (2008). Transmembrane proteins of tight junctions. Biochim. Biophys. Acta.

[12] Anderson JM, Van Itallie CM (2009). Physiology and function of the tight junction. Cold Spring Harbor Perspect Biol.

[13] Günzel D, Yu ASL (2013). Claudins and the modulation of tight junction permeability. Physiol. Rev..

[14] Oh J, Conlan S, Polley EC, Segre JA, Kong HH (2012). Shifts in human skin and nares microbiota of healthy children and adults. Genome Med..

[15] Grice EA, Segre JA (2011). The skin microbiome. Nat. Rev. Microbiol..

[16] Shu M, Wang Y, Yu J, Kuo S, Coda A (2013). Fermentation of propionibacterium acnes, a commensal bacterium in the human skin microbiome, as skin probiotics against methicillin-resistant *Staphylococcus aureus*. PLoS ONE.

[17] Wang Y (2014). Propionic acid and its esterified derivative suppress the growth of methicillin-resistant *Staphylococcus aureus* USA300. Benef. Microbes.

[18] Christensen GJM, Brüggemann H (2014). Bacterial skin commensals and their role as host guardians. Benef. Microbes.

[19] Pivarcsi A (2003). Expression and function of Toll-like receptors 2 and 4 in human keratinocytes. Int. Immunol..

[20] Nagy I (2005). Distinct strains of Propionibacterium acnes induce selective human β-defensin-2 and interleukin-8 expression in human keratinocytes through toll-like receptors. J. Invest. Dermatol..

[21] Megyeri K (2018). Propionibacterium acnes induces autophagy in keratinocytes: Involvement of multiple mechanisms. J. Invest. Dermatol..

[22] Szabó K (2017). Factors shaping the composition of the cutaneous microbiota. Br. J. Dermatol..

[23] O’Neill AM, Gallo RL (2018). Host-microbiome interactions and recent progress into understanding the biology of acne vulgaris. Microbiome.

[24] Young SK, Samuel BH (2010). Intestinal goblet cells and mucins in health and disease: Recent insights and progress. Curr. Gastroenterol. Rep..

[25] Allaire, J. M. *et al.* Erratum: The Intestinal Epithelium: Central Coordinator of Mucosal Immunity (Trends in Immunology (2018) 39(9) (677–696), (S1471490618300681), (10.1016/j.it.2018.04.002)). *Trends in Immunology* (2019). doi:10.1016/j.it.2018.12.008 10.1016/j.it.2018.04.002 29716793

[26] Srinivasan B (2015). TEER measurement techniques for in vitro barrier model systems. J. Lab. Autom..

[27] Benson K, Cramer S, Galla HJ (2013). Impedance-based cell monitoring: Barrier properties and beyond. Fluids Barriers CNS.

[28] Masago K (2018). Lysophosphatidic acid receptor, LPA6, regulates endothelial blood-brain barrier function: Implication for hepatic encephalopathy. Biochem. Biophys. Res. Commun..

[29] Bischoff I (2016). Pitfalls in assessing microvascular endothelial barrier function: Impedance-based devices versus the classic macromolecular tracer assay. Sci. Rep..

[30] Hennings H (1980). Calcium regulation of growth and differentiation of mouse epidermal cells in culture. Cell.

[31] Sun M (2012). A dynamic real-time method for monitoring epithelial barrier function in vitro. Anal. Biochem..

[32] Boyce ST, Ham RG (1983). Calcium-regulated differentiation of normal human epidermal keratinocytes in chemically defined clonal culture and serum-free serial culture. J. Invest. Dermatol..

[33] Kubo A, Nagao K, Yokouchi M, Sasaki H, Amagai M (2009). External antigen uptake by Langerhans cells with reorganization of epidermal tight junction barriers. J. Exp. Med..

[34] Nakamizo S (2015). Commensal bacteria and cutaneous immunity. Semin. Immunopathol..

[35] Brandner J (2014). Epidermal tight junctions in health and disease. Tissue Barriers.

[36] Stolwijk JA, Matrougui K, Renken CW, Trebak M (2015). Impedance analysis of GPCR-mediated changes in endothelial barrier function: overview and fundamental considerations for stable and reproducible measurements. Pflugers Arch. Eur. J. Physiol..

[37] Tax G (2016). Propionic acid produced by propionibacterium acnes strains contri—butes to their pathogenicity. Acta Derm. Venereol..

[38] Ohnemus U (2007). Regulation of epidermal tight-junctions (TJ) during infection with exfoliative toxin-negative *Staphylococcus* strains. J. Invest. Dermatol..

[39] Tan J (2014). The role of short-chain fatty acids in health and disease. Adv. Immunol..

[40] Günzel D (2017). Claudins: vital partners in transcellular and paracellular transport coupling. Pflugers Arch. Eur. J. Physiol..

[41] Mineta K (2011). Predicted expansion of the claudin multigene family. FEBS Lett..

[42] Capaldo CT, Nusrat A (2015). Claudin switching: Physiological plasticity of the tight junction. Semin. Cell Dev. Biol..

[43] Brandner JM (2002). Organization and formation of the tight junction system in human epidermis and cultured keratinocytes. Eur. J. Cell Biol..

[44] Atsugi T (2020). Holocrine secretion occurs outside the tight junction barrier in multicellular glands: Lessons from claudin-1–deficient mice. J. Invest. Dermatol..

[45] Matter K, Aijaz S, Tsapara A, Balda MS (2005). Mammalian tight junctions in the regulation of epithelial differentiation and proliferation. Curr. Opin. Cell Biol..

[46] Furuse M (2002). Claudin-based tight junctions are crucial for the mammalian epidermal barrier: A lesson from claudin-1-deficient mice. J. Cell Biol..

[47] Gruber R (2015). Diverse regulation of claudin-1 and claudin-4 in atopic dermatitis. Am. J. Pathol..

[48] Tokumasu R (2016). Dose-dependent role of claudin-1 in vivo in orchestrating features of atopic dermatitis. Proc. Natl. Acad. Sci. USA..

[49] Deng Z (2019). Claudin reduction may relate to an impaired skin barrier in rosacea. J. Dermatol..

[50] Li J, Li Q, Geng S (2019). All-trans retinoic acid alters the expression of the tight junction proteins Claudin-1 and-4 and epidermal barrier function-associated genes in the epidermis. Int. J. Mol. Med..

[51] Hatakeyama S, Ishida K, Takeda Y (2010). Changes in cell characteristics due to retinoic acid; specifically, a decrease in the expression of claudin-1 and increase in claudin-4 within tight junctions in stratified oral keratinocytes. J. Periodontal. Res..

[52] Erdei L (2018). TNIP1 regulates cutibacterium acnes-induced innate immune functions in epidermal keratinocytes. Front. Immunol..

[53] Leyden JJ, McGinley KJ, Mills OH, Kligman AM (1975). Propionibacterium levels in patients with and without acne vulgaris. J. Invest. Dermatol..

[54] Fitz-Gibbon S (2013). Propionibacterium acnes strain populations in the human skin microbiome associated with acne. J. Invest. Dermatol..

[55] Bienenfeld A, Nagler AR, Orlow SJ (2017). Oral antibacterial therapy for acne vulgaris: An evidence-based review. Am. J. Clin. Dermatol..

[56] Slanina H, König A, Claus H, Frosch M, Schubert-Unkmeir A (2011). Real-time impedance analysis of host cell response to meningococcal infection. J. Microbiol. Methods.

