# Supplementary material for: Cutibacterium acnes regulates the epidermal barrier properties of HPV-KER human immortalized keratinocyte cultures
Source: Sci Rep. 2020 Jul 30;10:12815. doi: 10.1038/s41598-020-69677-6 (PMC7393503; doi:10.1038/s41598-020-69677-6)
Supplement: Supplementary file 1 — Supplementary information. [file 41598_2020_69677_MOESM1_ESM.pdf]

***Cutibacterium acnes* regulates the epidermal barrier properties of HPV-KER human immortalized keratinocyte cultures**

Beáta Szilvia Bolla[1,2], Lilla Erdei[1,2], Edit Urbán[3], Katalin Burián[4], Lajos Kemény[1,2,5,#] and Kornélia Szabó [1,5,#,*]

1. Department of Dermatology and Allergology, University of Szeged, Szeged, Hungary

2. HCEMM-SZTE Skin Research Group, Szeged, Hungary

3. Department of Public Health, University of Szeged, Szeged, Hungary

4. Institute of Clinical Microbiology, University of Szeged, Szeged, Hungary

5. MTA-SZTE Dermatological Research Group, Szeged, Hungary

[#]The two senior authors contributed equally to the work.

*Correspondence

Kornélia Szabó
szabo.kornelia@med.u-szeged.hu

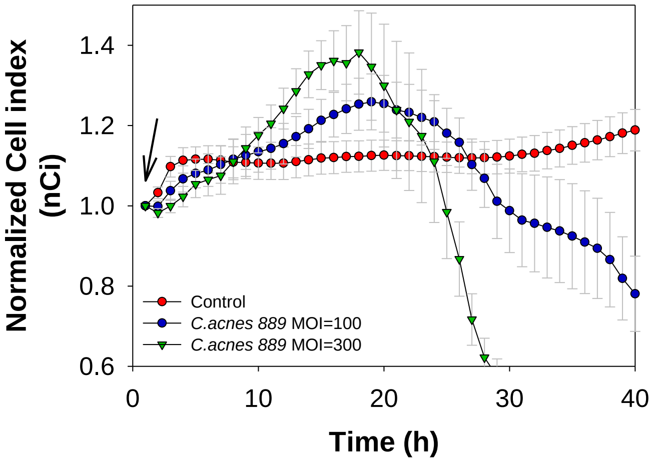

Supplementary figure 1. In Ca-low HPV-KER monolayer cultures, co-culturing with *C. acnes* 889 strain leads to transient, dose-dependent nCi increases characteristic of improved barrier functions of *in vitro* monolayer cultures. After the establishment of the Ca-low HPV-KER cultures, they were treated with different doses (MOI= 100, 300) of *C. acnes* 889 strain (0 hour time point, marked with an arrow). The transient, dose-dependent nCi increase observed is characteristic of improved barrier properties.

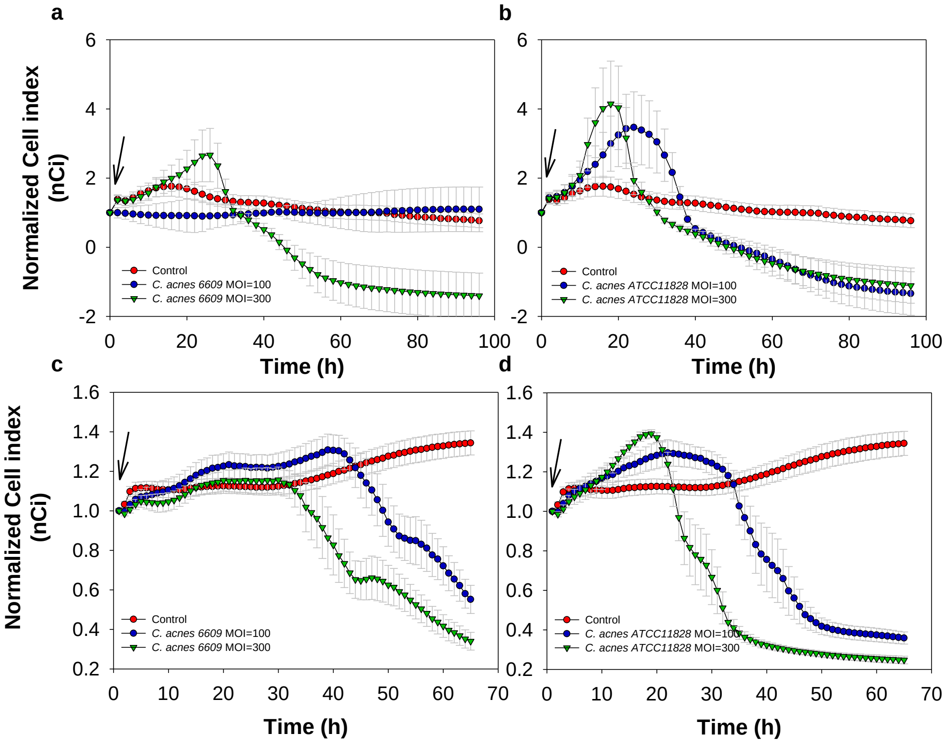

Supplementary figure 2. Different *C. acnes* strains have variable effects on the nCi values of NHEK and HPV-KER monolayer cultures. Ca-low NHEK (a, b) and HPV-KER (c, d) monolayer cultures were established and co-cultured with the *C. acnes* 6609 and *ATCC11828* strains (0 hour time point, marked with an arrow) in different doses (MOI= 100, 300). Both strains lead to transient nCi changes similar to those observed for the *C. acnes* 889 strain, but with altered kinetics.

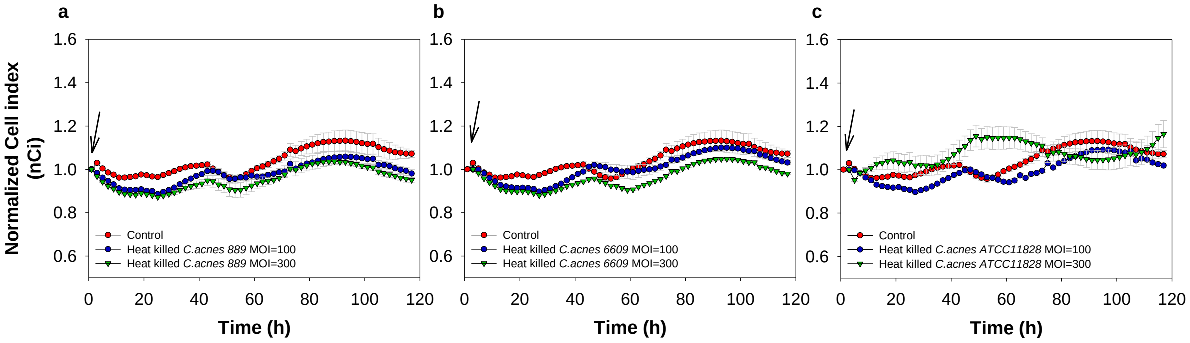

Supplementary figure 3. Heat-killed *C. acnes* strains do not have major effects on Ca-low HPV-KER monolayer cultures. After establishment of contact-inhibited monolayer cultures, they were treated with heat-killed *C. acnes 889* (a), *6609* (b) and *ATCC11828* (c) strains (0 hour time point, marked with an arrow) using different doses (MOI= 100, 300). No major changes in nCi values were detected.

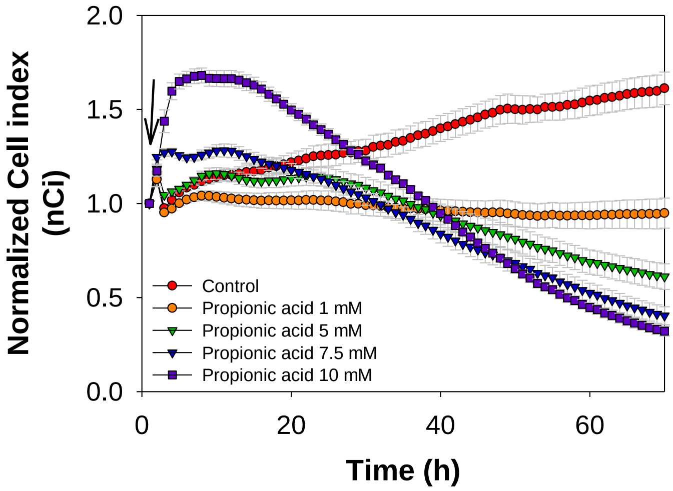

Supplementary figure 4. PA treatment modulates the nCi values of Ca-low HPV-KER monolayer cultures. Contact-inhibited monolayer cultures were treated with different doses of PA (1 mM, 5 mM, 7.5 mM, and 10 mM, 0 hour time point, marked with an arrow). Higher doses of PA treatment lead to transient nCi increases, characteristic of improved barrier properties of *in vitro* cultures.

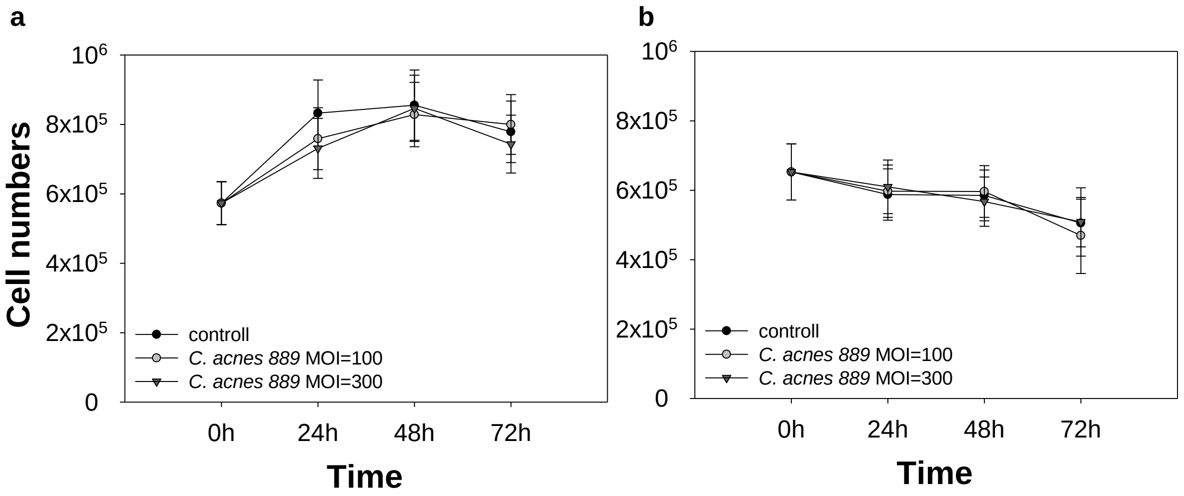

Supplementary figure 5. Cell numbers do not markedly change in Ca-low (a) and Ca-high (b) HPV-KER monolayers upon co-culturing with *C. acnes 889* strain. No major changes were detected in the number of cells in cultures after bacterial treatment, independent of the applied *C. acnes* doses. These data suggest that no major changes in the number of cells in the Ca-low and Ca-high HPV-KER cultures occurred after bacterial treatment. **(**Each treatment was performed in three technical replicates and data points represent the mean ± SEM.

Supplementary Materials and Methods:

Gene	Sequence	Probe number
18 S rRNA	F:5'CGCTCCACCAACTAAGAACG3'<br>R:5'CTCAACACGGGAAACCTCAC3'	77
Claudin 1 (CLDN1)	F: TTGACTCCTTGCTGAATCTGAG<br>R: GGCCACAAAGATTGCTATCAC	79
Claudin 4 (CLDN4)	F: TCACACCTGGGTCCCCTA<br>R: TCGCTTTAACCTGGGAGATG	19
Occludin (OCLN)	F: GTCATCCAGGCCTCTTGAAA<br>R: GGTGCATAATGATTCGGTTTG	10
Zonula occludens 1 (ZO-1)	F: TCAGACAGGCGGTCAGTG<br>R: ATATGGCTTGCCAATCGAAG	20

Supplementary table 1. List of the used primers and probes.

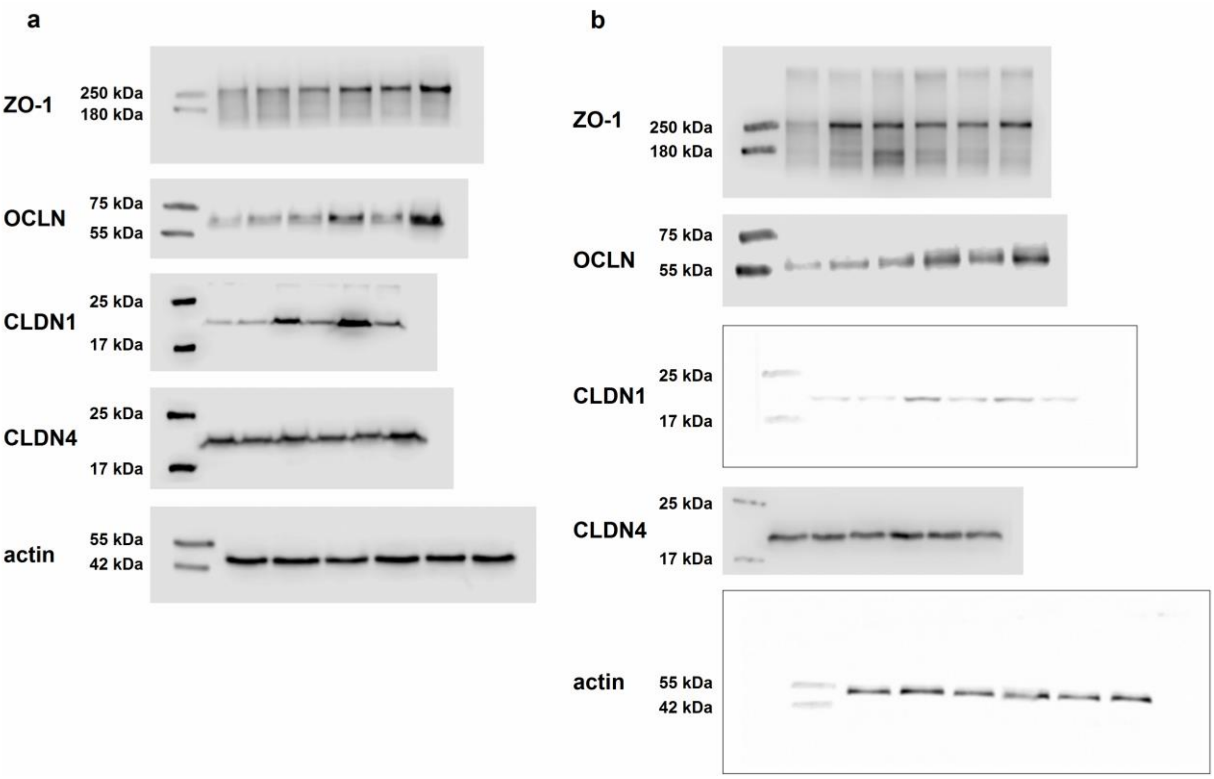

Supplementary Figure 6. Original western blots of Ca-low (a) and Ca-high (b) HPV-KER cultures co-cultured with *C. acnes* 889 strain. Western blot membranes were cropped using the protein marker as a guide. Next, specific antibody or antibodies were applied for each half to detect the protein of interest, represented by the particular piece. CLDN1, 4 and the loading control actin were assayed using the same blot, which we cut slightly higher than

the 25 kDa marker lane. In the case of actin, the upper part was probed, where the bands were visible approximately at 40 kDa. The lower part was probed first for CLDN 4, and CLDN 1 was detected after stripping. ZO-1 and OCLN were assayed on a different blot, which was prepared parallel to the first one using the same protein amounts for each lane as in the previous blot. In this case, the membrane was cut between 95 kDa and 100 kDa. ZO-1 was probed for the part representing proteins above 100 kDa, and specific bands appeared around 200 kDa. The other piece was used to detect OCLN, where the bands appeared approximately at 60 kDa. Western blots representing CLDN1 and actin expression in Ca-high cultures were visualized by the Omega Lum G Chemidoc Imageing System. For all the other blots, C-DiGit Blot Scanner was applied.

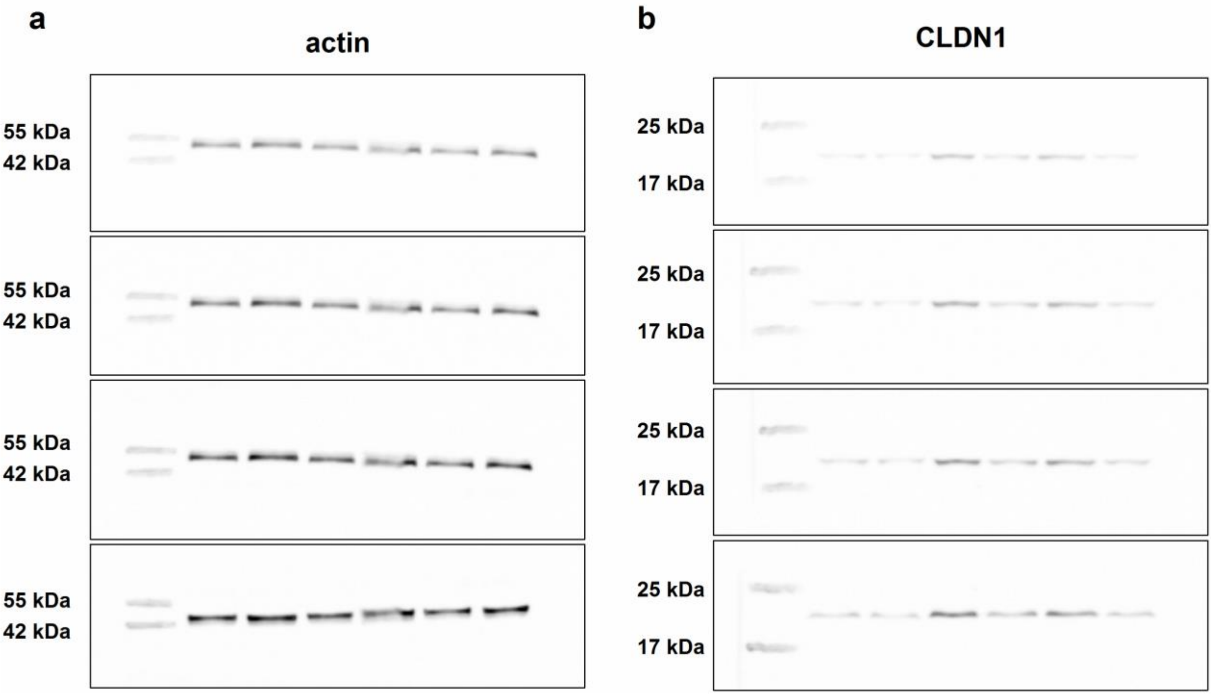

Supplementary Figure 7. Original actin (a) and CLDN1 (b) western blots of Ca-high HPV-KER cultures upon treatment with *C. acnes 889* strain, representing different exposure times.
